# Editorial for Oral Microbes and Human Health

**DOI:** 10.3390/microorganisms13040922

**Published:** 2025-04-16

**Authors:** Andrea Butera, Andrea Scribante

**Affiliations:** 1 Unit of Dental Hygiene, Section of Dentistry, Department of Clinical, Surgical, Diagnostic and Pediatric Sciences, University of Pavia, 27100 Pavia, Italy; 2 Unit of Orthodontics and Pediatric Dentistry, Section of Dentistry, Department of Clinical, Surgical, Diagnostic and Pediatric Sciences, University of Pavia, 27100 Pavia, Italy

The human oral cavity harbors a complex and dynamic microbial ecosystem, comprising over 700 bacterial species, alongside fungi, viruses, and protozoa [1]. This intricate community, known as the oral microbiome, plays a pivotal role in maintaining oral and systemic health. Disruptions in this delicate balance can lead to oral diseases such as dental caries and periodontal disease, which have been linked to broader systemic conditions, including cardiovascular diseases, diabetes, and respiratory infections [2]. Understanding the interplay between oral microbes and human health is essential for developing effective prevention and treatment strategies.

The oral microbiome is a diverse assembly of microorganisms that colonize various surfaces within the mouth, including teeth, gums, tongue, and mucosal membranes [3]. In a state of equilibrium, these microbes contribute to oral health by inhibiting the colonization of pathogenic species through competitive exclusion and the production of antimicrobial substances. For instance, commensal bacteria such as Streptococcus sanguinis and Streptococcus gordonii produce hydrogen peroxide, which suppresses the growth of pathogens like Porphyromonas gingivalis and Aggregatibacter actinomycetemcomitans, known contributors to periodontal disease [4].

When the balance of the oral microbiome is disrupted—a condition known as dysbiosis—opportunistic pathogens can proliferate, leading to oral diseases [5]. Periodontal disease, characterized by inflammation of the supporting structures of the teeth, is a prime example. It results from the accumulation of pathogenic biofilms that elicit an inflammatory response, causing tissue destruction and bone loss [6]. Notably, periodontal disease has been associated with systemic conditions such as cardiovascular diseases and diabetes, highlighting the far-reaching implications of oral microbial imbalances [7].

The use of probiotics—live microorganisms that confer health benefits to the host—has emerged as a promising adjunct in managing oral health [8]. Probiotics can modulate the oral microbiome by competing with pathogenic bacteria for adhesion sites and nutrients, producing antimicrobial compounds, and enhancing the host’s immune response [9]. Studies have shown that probiotic strains like Lactobacillus reuteri and *Lactobacillus brevis* can reduce periodontal pocket depth and improve clinical attachment levels in patients with periodontal disease [10]. However, the efficacy of probiotics can vary, and their benefits are often strain-specific [11].

Probiotics exert their beneficial effects through multiple mechanisms that collectively contribute to oral health. One of the key strategies involves competitive exclusion, where probiotic bacteria compete with pathogenic microorganisms for adhesion sites on oral tissues, effectively limiting the ability of harmful species to establish themselves. Additionally, certain probiotic strains produce antimicrobial substances, such as bacteriocins, that directly inhibit the growth of pathogenic bacteria, further reducing their presence in the oral cavity. Beyond their antimicrobial properties, probiotics play a role in modulating the host’s immune response by stimulating the production of anti-inflammatory cytokines while simultaneously downregulating pro-inflammatory mediators, thereby reducing excessive inflammation associated with oral diseases. Moreover, probiotics influence the local pH of the oral environment by producing acids or alkali, altering conditions to make them less favorable for the survival and proliferation of pathogenic bacteria. Through this multifaceted approach, probiotics help restore and maintain a balanced oral microbiome, ultimately promoting overall oral health [12].

Clinical trials have investigated the role of probiotics in managing various oral conditions. Research has demonstrated that probiotics can contribute to periodontal health by reducing gingival inflammation, minimizing bleeding on probing, and decreasing periodontal pocket depths [13]. Probiotic based interventions led to improved periodontal outcomes, characterized by decreased bleeding and modifications in the composition of oral bacteria [14]. In addition to their impact on periodontal disease, probiotics have shown potential in reducing the risk of dental caries by inhibiting the growth of cariogenic bacteria such as *Streptococcus mutans* [15,16,17,18]. However, studies on this subject have produced mixed results, and additional research is required to establish definitive conclusions regarding their efficacy [19]. Furthermore, probiotics have been explored as a remedy for halitosis, as certain strains can reduce volatile sulfur compounds responsible for bad breath, thus contributing to fresher breath and improved oral hygiene [20].

The oral microbiome plays a critical role in maintaining both oral and systemic health. Dysbiosis within this microbial community can lead to periodontal disease and other oral conditions, with potential systemic implications. Probiotics offer a promising avenue for modulating the oral microbiome, thereby contributing to the prevention and management of oral diseases. However, further research is needed to fully elucidate their mechanisms of action, optimize delivery methods, and confirm their long-term efficacy and safety. Integrating probiotics into oral healthcare regimens could represent a significant advancement in promoting overall health and well-being.

This Special Issue thus explores new strategies and possibilities in the exciting field of oral and systemic health through investigations in three research papers (contributions 1 to 3) and seven review manuscripts (contributions 4 to 10).
